# Unplanned pregnancy and the association with maternal health and pregnancy outcomes: A Swedish cohort study

**DOI:** 10.1371/journal.pone.0286052

**Published:** 2023-05-22

**Authors:** Alisa Carlander, Jenny Niemeyer Hultstrand, Isa Reuterwall, Maria Jonsson, Tanja Tydén, Merit Kullinger

**Affiliations:** 1 Department of Obstetrics and Gynecology, Region Västmanland, Västerås, Sweden; 2 Department of Women´s and Children’s Health, Uppsala University, Uppsala, Sweden; University of Tennessee Knoxville, UNITED STATES

## Abstract

**Objectives:**

Unplanned pregnancies are common and associated with late initiation and inadequate antenatal care attendance, which may pose health risks to mother and child. How pregnancy planning relates to maternal health and delivery in Sweden, a country with free antenatal care and free abortion, has not been studied previously. Our aims were to study whether pregnancy planning was associated with antenatal care utilization and pregnancy outcomes in a Swedish setting.

**Methods:**

Data for 2953 women, who answered a questionnaire when recruited at antenatal clinics in Sweden and later gave birth, was linked to the Swedish Medical Birth Register. The degree of pregnancy planning was estimated using the London Measure of Unplanned Pregnancy. Unplanned (comprising unplanned and ambivalent intention to pregnancy) was compared to planned pregnancy. Differences between women with unplanned and planned pregnancy intention and associated pregnancy outcomes were analyzed using Fisher’s exact test and logistic regression.

**Results:**

There were 31% unplanned (2% unplanned and 29% ambivalent) pregnancies, whereas most woman (69%) reported their pregnancy to be planned. Women with an unplanned pregnancy enrolled later to antenatal care, but there was no difference in number of visits compared with planned pregnancy. Women with an unplanned pregnancy had higher odds to have induced labor (17% versus 13%; aOR 1.33 95% CI 1.06–1.67) and a longer hospital stay (41% versus 37%; aOR 1.21 95% CI 1.02–1.44). No associations were found between pregnancy planning and pregnancy-induced hypertension, gestational diabetes mellitus, preeclampsia, epidural analgesia use, vacuum extraction delivery, Caesarean section or sphincter rupture.

**Conclusions:**

Unplanned pregnancy was associated with delayed initiation of antenatal care, higher odds for induction of labor and longer hospital stay, but not with any severe pregnancy outcomes. These findings suggest that women with an unplanned pregnancy cope well in a setting with free abortion and free health care.

## Introduction

Unplanned pregnancies are common worldwide. Approximately, 40% of the 213 million pregnancies occurring in 2012 were unplanned and 38% resulted in a birth [1]. Unplanned pregnancy is associated with late initiation and inadequate use of antenatal care [2, 3] and, in some settings, with adverse neonatal outcomes such as low birth weight and preterm birth [4, 5]. Both in the US and Europe, women with unplanned pregnancies are more often young, single or have a non-cohabiting relationship, are foreign born, have a low educational level [6, 7] and higher parity [8, 9]. Also, exposure to intimate partner violence is associated with having an unplanned pregnancy [8, 10].

All women in Sweden are offered free healthcare during pregnancy and childbirth, including access to health surveillance and antenatal care provided by midwives and specialist care provided by obstetricians. Almost all women who continue with a pregnancy attend an antenatal clinic and more than 95% visit a midwife regularly [11]. However, if the pregnancy was initially planned or not is not routinely assessed. In a study by Stern et al, 12% of the pregnancies in Sweden were very or fairly unplanned [12].

The number of unplanned pregnancies that also lead to an unplanned birth will vary as women of different countries will face differences in legislation and abortion care facilities. The Swedish Abortion Act from 1975 (SFS 1974:595) allows a woman to have an abortion at her own request and free of cost until gestational week 18 + day 0; and later abortion can in some cases be approved by the National Board of Health and Welfare [13]. In Sweden, the abortion rate is among the highest in Europe [14] at 18 per 1000 women aged 15–44 years [15]. Most abortions (61%) are completed before the 7^th^ gestational week [15].

Knowledge on how an unplanned pregnancy intention impacts on child and maternal health on short and long term is limited. Women with unplanned, compared to planned pregnancy, report higher anxiety levels during pregnancy [16], experience more negative feelings and perceive more pain during labor [17]. Women with unplanned pregnancy also have higher levels of depressive symptoms before pregnancy [18] and in the perinatal period [19, 20], and are also at risk for postpartum depression [21, 22]. It has been suggested that a less planned pregnancy is associated with fear of labor and higher levels of Cesarean section requests [23] as well as induction of labor [24]. It is unclear if induction rates, for medical or psychiatric reasons, are higher in unplanned compared to planned pregnancy.

Attempts to study pregnancy intention are often limited by methodological issues, such as a retrospective study design, sometimes up to 5 years after giving birth [2], and/or using only a single question [3, 25]. In 2004 [26], the London Measure of Unplanned Pregnancy (LMUP) was developed to define and assess degrees of pregnancy planning [27]. The LMUP is a psychometrically validated instrument [27] that is used increasingly worldwide and has been validated in several countries and in different languages [28–35].

The aims of the current study were to assess whether unplanned pregnancy, in a Swedish setting, was associated with lower compliance to antenatal care and if women with unplanned pregnancy intention had higher odds for adverse outcomes during pregnancy and labor.

## Methods

This observational study was based on survey data retrieved from the cross-sectional Swedish Pregnancy Planning cohort study described by Stern et al [12] and register data from the Swedish Medical Birth Register (MBR). Data linkage was performed by the National Board of Health and Welfare and was enabled through use of the personal identification number. The data were de-identified.

Recruitment to the study took place between September 2012 and July 2013. In 10 Swedish counties, 5494 women were asked to participate when registering in an antenatal care unit (ACU). A detailed flow chart of the study population is presented by Stern et al, including reasons why women were not asked to participate or declined participation [12]. Women entered the study at their first antenatal visit, usually around 10^th^ gestational week, by completing a questionnaire comprising 148 items covering background characteristics, planning of the current pregnancy, physical and mental health, sexual and reproductive health, lifestyle, and partnership (if any) [12].

The degree of pregnancy planning was assessed using the LMUP, which comprises six questions, each of which is scored from 0 to 2. The scores are added to give a sum from 0 to 12, where higher scores represent a higher degree of pregnancy planning. The researchers who developed the LMUP emphasize that there are no obvious cutoffs in the scale, but that each score provides important information. However, three groups are defined for population estimates: unplanned pregnancy (score 0–3), ambivalent intention about the pregnancy (score 4–9), and planned pregnancy (score 10–12) [26]. In this study the women were divided into two subgroups based on the scores: planned pregnancy (ten points or more) and unplanned pregnancy (up to nine points). Hall et al recommend merging the ambivalent and unplanned group to one unplanned group when using the LMUP scores in logistic regression analysis [27].

Data from the MBR were retrieved for all participants whose pregnancy resulted in a birth. The MBR started in 1973 and contains data on more than 98% of all births in Sweden. Data from antenatal, obstetric, and neonatal care, as well as the diagnoses according to the International Classification of Diseases relating to complications during pregnancy, delivery, and perinatal period are collected prospectively and recorded in the MBR [36]. The quality of data is considered to be high and reliable for research purposes [37].

### Definitions of outcomes and variables

Background characteristics were mainly based on self-reported information from the questionnaire and included degree of pregnancy planning, age, pre-pregnancy maternal weight, height, educational level, country of birth, parity, family situation, pre-pregnancy folic acid use, smoking, alcohol use, medical history and late detection of pregnancy. Some background variables were also possible to retrieve from the MBR, but, for consistency, data from the questionnaire were used except for the total number of visits to the ACU that had to be retrieved from the MBR.

Body mass index (BMI) was calculated as pre-pregnancy weight in kilograms divided by the square of height in meters. The educational level, which was used as a proxy for socioeconomic status [38], was categorized according to the International Standard Classification of Education (ISCED) [39]. Three levels were defined: low (maximum 9 years of education, ISCED codes 0–2), medium (maximum 12 years of education, ISCED codes 3 and 4), and high (more than 12 years of education, ISCED codes 5–8) [40]. Parity was grouped into nullipara and parous. Women were asked about medical conditions before pregnancy by choosing among a set number of options or, when not applicable, written in free text. The time of awareness of the pregnancy could be chosen between nine different options of gestational weeks from “2^nd^ or earlier” up to “10^th^ or later”. We defined this as “early recognition” before the 10^th^ gestational week and “late awareness” at the 10^th^ gestational week or later.

The studied outcomes during pregnancy and labor were variables retrieved from the MBR and included low utilization (0–6 visits) or high utilization (≥12 visits) of antenatal care (the median value for both groups ±2 visits was used as the reference value in the analyses), the presence of pregnancy-induced hypertension, gestational diabetes mellitus, or preeclampsia, labor pain relief (morphine administration or epidural anesthesia), induction of labor, operative delivery (vacuum extraction or Cesarean section), sphincter rupture, postpartum hemorrhage, and duration of hospital stay (calculated as the difference between the date of admission and the date of discharge as registered in the MBR).

### Statistical analyses

Categorical data are presented as number and percentage and groups were compared using Fisher’s exact test. Logistic and multinomial regression analyses were used to analyze maternal outcomes. Covariates were chosen after using DAGs [41] in the process of identifying plausible confounders for the respective outcomes (S1–S8 Files). Psychiatric illness was not considered a confounder but rather an effect moderator.

For the statistical analyses, the score on the LMUP was used as a dichotomous variable to identify potential differences between planned and unplanned pregnancies. A cutoff score of 9 was used: scores 0–9 were classified as an unplanned and ≥10 as a planned pregnancy [27]. Missing values were imputed as instructed by Barret et al [26]. For all statistical analyses, a p value < 0.05 was considered to be significant. The data were entered and analyzed using IBM SPSS Statistics (version 25).

### Ethics

The Regional Ethical Review Board in Uppsala, Sweden, approved the study protocols (reference numbers 2010/085, 2010-06-01 and 2017/085/5, 2017-07-20). Return of the completed questionnaire was regarded as informed consent.

## Results

**Fig 1:**
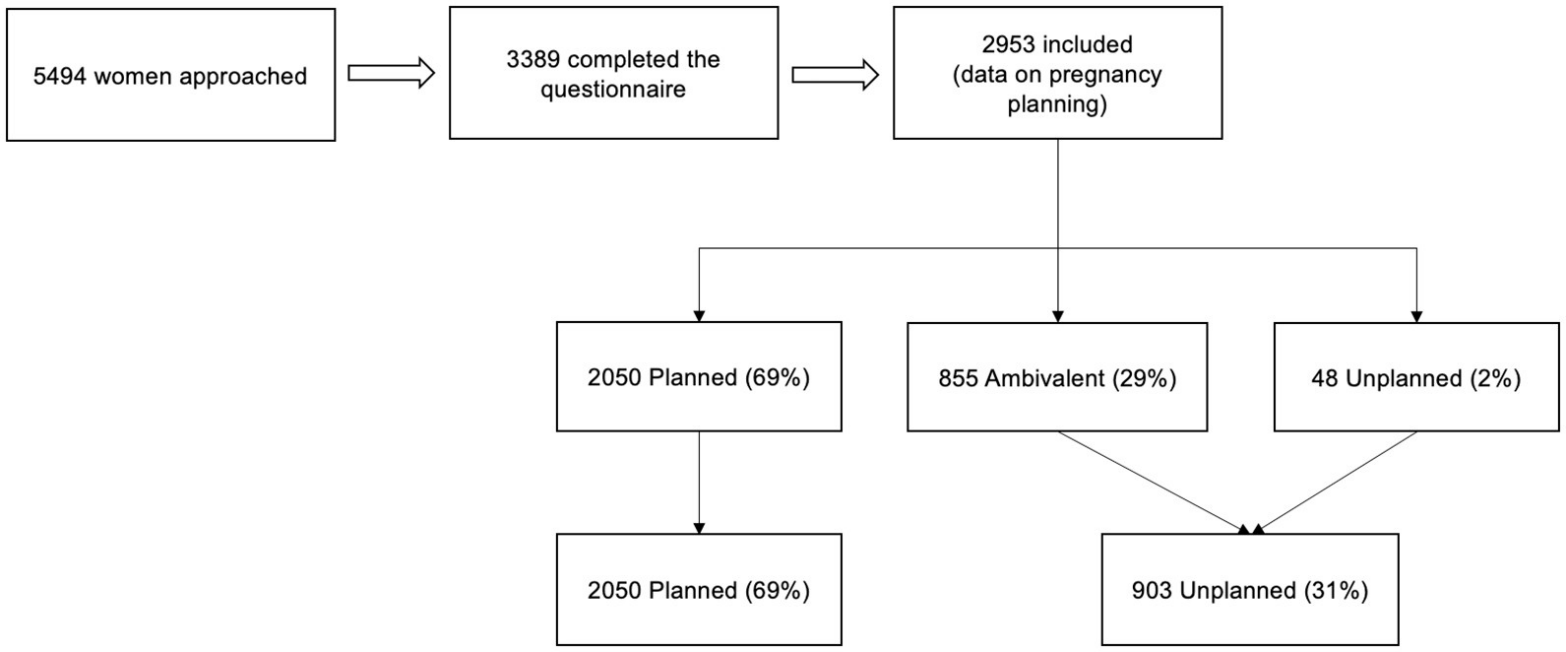
Flowchart of the study population.

In total, 5494 women were invited to participate, 4969 women accepted participation and, in total, 3389 women were included upon completing the questionnaire (Fig 1). Of the included women, 98% used the questionnaire in Swedish, 1% used a translated questionnaire (English or Arabic), and 1% were interviewed by telephone by an interpreter (all other languages). Non-response to individual questions was only 0.3–5%, hence missing values were disregarded. The majority, 2953 women (87%), answered the questions on pregnancy planning, and 31% defined their intention as unplanned (2% unplanned and 29% ambivalent) whereas 69% as planned. Women with an unplanned pregnancy that continued their pregnancy to birth were younger, more often foreign-born, single, and had a lower educational level compared with women with a planned pregnancy (Table 1). Even so, 94% of the women with an unplanned pregnancy carried to birth had reached a medium or high educational level.

**Table 1:** Background characteristics of women with an unplanned (n = 903) or planned (n = 2050) pregnancy presented as number and percentage.

	Total		Unplanned		Planned		p-value ^a^
	n	%	n	%	n	%	
**Maternal age (years)**							
<21	88	3	52	6	36	2	<0.001
21–25	575	20	220	25	349	18	<0.001
26–35	1897	66	487	56	1386	70	<0.001
>35	338	12	115	13	217	11	0.100
**BMI (kg/m** ^**2**^ **)**							
<18.5	96	3	34	4	62	3	0.309
18.5–24.9	1809	64	543	63	1266	64	0.581
25–29.9	614	22	192	22	422	21	0.586
≥30	318	11	93	11	225	12	0.698
**Education (years)**							
<9	186	6	92	10	94	5	<0.001
9–12	1133	39	397	45	736	37	<0.001
>12	1582	55	399	45	1183	59	<0.001
**Foreign origin**	331	11	127	14	204	10	<0.001
**Parity**							
Nulliparous	1329	45	420	47	909	44	0.279
Parous	1600	54	474	52	1126	55	0.229
**Partner at the first visit to the ACU**	2909	99	869	97	2040	99.8	<0.001
**Use of folic acid 1 month before pregnancy**	959	33	79	9	880	43	<0.001
**Smoking 3 months before pregnancy**	593	20	267	30	326	16	<0.001
**Smoking at the first visit to the ACU**	127	4	77	9	50	3	<0.001
**High alcohol use 3 months before pregnancy** ^b^	111	5	56	8	55	3	<0.001
**Any alcohol use at the time of the first visit to the ACU**	12	0.4	4	0.4	8	0.4	0.763
**History of psychiatric illness**	284	10	114	13	170	8	<0.001
**History of somatic illness**	1231	42	383	42	848	41	0.599
**Number of visits to the ACU**							
0–7	568	22	185	23	383	19	0.256
≥8	2064	78	621	77	1443	71	0.256
**Late detection of pregnancy** ^c^	36	1	26	3	10	1	<0.001

^a^ Fisher’s exact test

^b^ High alcohol use defined as >5 standard glasses of alcohol/week.

^c^ Late detection of pregnancy defined as gestational week 10 or later.

ACU: Antenatal care unit, BMI: body mass index.

Women with an unplanned pregnancy used folic acid less often in the month before they became pregnant, had higher rates of smoking during pregnancy and were more likely to have high alcohol consumption three months prior to pregnancy, which has been previously reported [12]. Having a history of psychiatric illness was more frequently reported by women with an unplanned, compared to planned, pregnancy intention. The number of ACU visits did not differ significantly between women with an unplanned or planned pregnancy, and both groups reached the recommended eight visits during pregnancy (Table 1).

Women with an unplanned pregnancy that continued their pregnancy to birth had higher odds to have induced labor (17% versus 13%; aOR 1.33 95% CI 1.06–1.67), and a hospital stay longer than 3 days (41% versus 37%; aOR 1.21 95% CI 1.02–1.44) compared with those with a planned pregnancy (Table 2). Pregnancy planning was not significantly associated with higher odds for pregnancy-induced hypertension, gestational diabetes mellitus, preeclampsia, vacuum extraction delivery, Cesarean section (elective or emergency), epidural analgesia use, or sphincter rupture.

**Table 2:** Maternal outcomes according to the degree of pregnancy planning.

	Total		Unplanned		Planned		OR	(95% CI)	p value	aOR^a^	(a95% CI)^a^	ap value^a^
	n	%	n	%	n	%						
**Utilization of antenatal care**												
Low use (0–6 visits)^b^	260	12	97	15	163	11	1.42	(1.09–1.87)	0.010	1.31	(0.99–1.73)	0.061
High use (≥12 visits)^b^	516	22	162	22	354	21	1.10	(0.89–1.35)	0.399	1.04	(0.84–1.30)	0.717
**Complications during pregnancy**												
Pregnancy-induced hypertension^c^	133	5	43	5	90	5	1.10	(0.76–1.26)	0.625	1.15	(0.78–1.68)	0.481
Gestational diabetes mellitus^c^	52	2	15	2	37	2	0.93	(0.51–1.70)	0.802	1.02	(0.55–1.89)	0.955
Preeclampsia^c^	138	5	47	6	91	5	1.19	(0.83–1.71)	0.342	1.08	(0.74–1.59)	0.693
**Intrapartum analgesia**												
Epidural analgesia^d^	956	32	301	33	655	32	1.07	(0.90–1.26)	0.460	1,05	(0.88–1.25)	0.602
Morphine, parenteral administration^d^	69	2	29	3	40	2	1.67	(1.03–2.71)	0.037	1.60	(0.99–2.610)	0.06
**Induced labor** ^e^	416	14	150	17	266	13	1.3	(1.08–1.66)	0.009	1.33	(1.06–1.67)	**0.015**
**Mode of delivery** ^e^												
Vacuum extraction^e^^,^^f^	206	7	53	6	153	8	0.77	(0.55–1.08)	0.126	0.82	(0.58–1.17)	0.280
Cesarean section^e^^,^^g^	499	18	150	18	349	19	0.96	(0.77–1.18)	0.683	1.02	(0.82–1.28)	0.830
**Complications during delivery**												
Sphincter rupture^c^	46	2	11	1	35	2	0.71	(0.36–1.40)	0.323	0.70	(0.34–1.43)	0.326
Postpartum hemorrhage^e^	48	1,8	12	1,5	36	1,9	0.76	(0.39–1.47)	0.410	1.00	(0.51–1.97)	0.993
Hospital stay ≥3 days^h^	1029	38	339	41	690	37	1.20	(1.02–1.42)	0.030	1.21	(1.02–1.44)	**0.034**

^a^ Adjusted OR, 95% CI, and p value.

^b^ Adjusted for maternal age, low educational level (maximum 9 years) and foreign origin.

^c^ Adjusted for maternal age, BMI (<18,5 or ≥ 30), low educational level (maximum 9 years) and foreign origin.

^d^ Adjusted for being parous and not living with partner.

^e^ Adjusted for maternal age, BMI (<18,5 or ≥ 30) and low educational level (maximum 9 years).

^f^ Compared to women who were not induced (i.e., spontaneous labor, planned and emergency Cesarean section).

^g^ Compared to vaginal non-instrumental birth

^h^ Adjusted for maternal age, BMI (<18,5 or ≥ 30), low educational level (maximum 9 years) and not living with partner.

OR: odds ratio, CI: confidence interval

## Discussion

Women with an unplanned pregnancy or an ambivalent intention to become pregnant accounted for nearly one-third of all pregnancies in the current study. Women with an unplanned pregnancy that resulted in birth had higher odds for having an induced labor and a longer hospital stay, but no increased odds for severe complications related to pregnancy and labor, when compared to women with a planned pregnancy. Women with an unplanned pregnancy were also more likely to have a late detected pregnancy (defined as after 10^th^ gestational week), lower socioeconomic status, be foreign-born, and have a history of psychiatric illness.

We found that only 2% of the pregnancies were completely unplanned, and this proportion is similar to that reported in studies from Belgium [8], Denmark [42], and Australia [33] that also used the LMUP to assess pregnancy planning. The prevalence is higher in other studies and may reflect the use of different or unvalidated instruments to measure pregnancy planning or different settings, including different countries and at what time point during pregnancy the woman was approached [1, 5, 43]. In the current study, unplanned pregnancies leading to abortions were not included, thus the overall prevalence of unplanned pregnancy is likely to be higher [44].

In the present study, the first visit to the ACU usually occurred in the 10^th^ gestational week and the vast majority (99% of the total study population) had an early recognition of the pregnancy (before 10^th^ gestational week). The World Health Organization recommends a minimum of eight visits to the ACU, with the first contact no later than gestational week 12, to reduce perinatal mortality [45]. Our finding that women with an unplanned pregnancy enrolled later in the ACU is consistent with earlier studies [2, 3, 8]. It is important to point out that the women in our study were approached at the ACU, and thus maybe already starting to accept the pregnancy, which seems to be of greater importance for the outcome than the planning status itself [9]. Once the women with an unplanned pregnancy were enrolled in the ACU, there was an equal probability of attaining the recommended number of visits as for women with a planned pregnancy. These findings support the results by Kost et al by suggesting that a woman’s behavior changes as soon as the pregnancy is recognized [46] and that women with an unplanned pregnancy can cope well in a setting that provides support in cases of the pregnancy continuing to birth.

To our knowledge, this is the first study to report increased odds ratios for induced labor among women with an unplanned pregnancy. In Sweden, there are national recommendations on induction of labor related to some medical conditions such as preeclampsia and prolonged pregnancy, although decisions on implementation and capacity to fulfil these recommendations can vary between different counties [47, 48]. Unfortunately, we had no information about the indications for induction of labor in this study. The incidence of pregnancy-induced medical complications did not differ between the groups. However, there was a significantly higher rate of reported psychiatric illness among women with an unplanned pregnancy. According to a literature review from Spada et al, there are few studies exploring induction of labor in women with psychiatric conditions [49]. In a Swedish study by Sydsjö et al, induction of labor was more frequent in women with fear of childbirth, a group of women that are characterized by a lower degree of planned pregnancies [24]. Further, women with a psychiatric vulnerability have an overall higher risk of unplanned pregnancy compared to women without a psychiatric vulnerability [50]. In addition to this, unplanned pregnancy is associated with factors, such as economical strain, being single or being in a violent relationship, that could predispose for depression [51]. In summary, there are several factors suggesting that women with unplanned pregnancy that continue to birth experience more stress and/or have a psychiatric illness that could lead to a request of induction of labor. These factors can also be a reason for the higher odds for longer hospital stay in women with unplanned pregnancy, which to our knowledge has not been studied before, as a history of psychiatric illness or a perceived vulnerability can be reasons for not recommending early discharge after delivery. We chose not to adjust the models for psychiatric illness as it was not considered a confounder, however it would be suspected to act as a moderator regarding both induction of labor and hospital stay.

An important finding is the lack of associations between pregnancy planning and several maternal health outcomes. Women who choose to continue an unplanned pregnancy to birth in this study did not differ regarding most outcomes, possibly because of the high accessibility to safe abortion, which provides a choice about continuing the pregnancy or not within the time frame of the law. As mentioned above, this lack of associations between planning and maternal health outcomes may also be an indication of behavioral changes, such as following health recommendations during pregnancy, once the decision to keep the pregnancy is made. In this study, the vast majority of women decided to enroll for ANC before 10^th^ gestational week, and, since they reached the recommended number of ACU visits we assume they got sufficient support. Interestingly, most women with an unplanned pregnancy had a medium or high educational level, interpreted as a proxy for medium and high socioeconomic level acting as a protecting factor, which might be over-represented in our study and contribute to the lack of adverse outcomes [52, 53]. In addition, Sweden is known for providing a generally high welfare level, which may have contributed to these results.

### Strengths and limitations

This was one of the first studies on unplanned pregnancy and pregnancy outcomes based on a validated instrument to assess pregnancy planning. In contrast to most earlier studies, women answered the question about pregnancy planning early in pregnancy, which reduced the risk of recall bias. Another strength was that women were recruited from 10 different regions in Sweden, which increases generalizability. The questionnaire data has earlier been compared to two national registers (the Swedish Medical Birth Register and the Swedish Pregnancy Register) to assess the generalizability [54]. The combination of self-reported data and data extracted from medical registers allowed for more analyses than a solely register-based study.

A limitation of our study was that pregnancy planning was used as a dichotomous variable (unplanned/ambivalent in relation to planned) instead of creating three groups (planned, ambivalent, and unplanned) or as a continuous variable. The rationale for using two groups was the small number of unplanned pregnancies. A continuous variable should have had greater statistical power than a dichotomous variable, but the dichotomous variable was considered to have greater clinical value. Another limitation was that the study was not designed to distinguish between early and late detection of pregnancy (i.e., after the limit for free abortion at gestational week 18), which may have provided further information about pregnancy outcomes related to women with very late detected pregnancies, however few. Also, the generalizability of the results is limited to countries with similar general healthcare and abortion services. In Sweden, the prevalence of unplanned pregnancies would be expected to be higher if women were approached before the 7^th^ gestational week in a gynecological out-patient department where abortion services are provided than in an ANC unit where women enroll for maternal health care, as in the current study. Also, we do not have data on the women who decided to terminate their pregnancy or had a miscarriage after ANC enrollment. Further, 98% of women completed the questionnaire in Swedish indicating that women of foreign origin without sufficient Swedish knowledge were under-represented in our data.

The prevalence of these outcomes were high, thus the ORs cannot be interpreted as risk ratios. However, logistic regression and OR is frequently used in clinical studies and well understood.

## Conclusions

Approximately one in three women had births stemming from either an unplanned or ambivalent intention to pregnancy in this setting. These women had higher odds for induction of labor and longer hospital stay compared with those with a planned pregnancy. However, the odds for the majority of maternal health and pregnancy outcomes did not differ regardless of whether the pregnancy was unplanned or planned, which may reflect the social context in a developed country such as Sweden with the benefit of free health care and free abortion.

## Supporting information

S1 File (PDF)

S2 File (PDF)

S3 File (PDF)

S4 File (PDF)

S5 File (PDF)

S6 File (PDF)

S7 File (PDF)

S8 File (PDF)

S9 File (PDF)
