# Supplementary figures and images for: Unplanned pregnancy and the association with maternal health and pregnancy outcomes: A Swedish cohort study
Source: PLoS One. 2023 May 22;18(5):e0286052. doi: 10.1371/journal.pone.0286052 (PMC10202275; doi:10.1371/journal.pone.0286052)

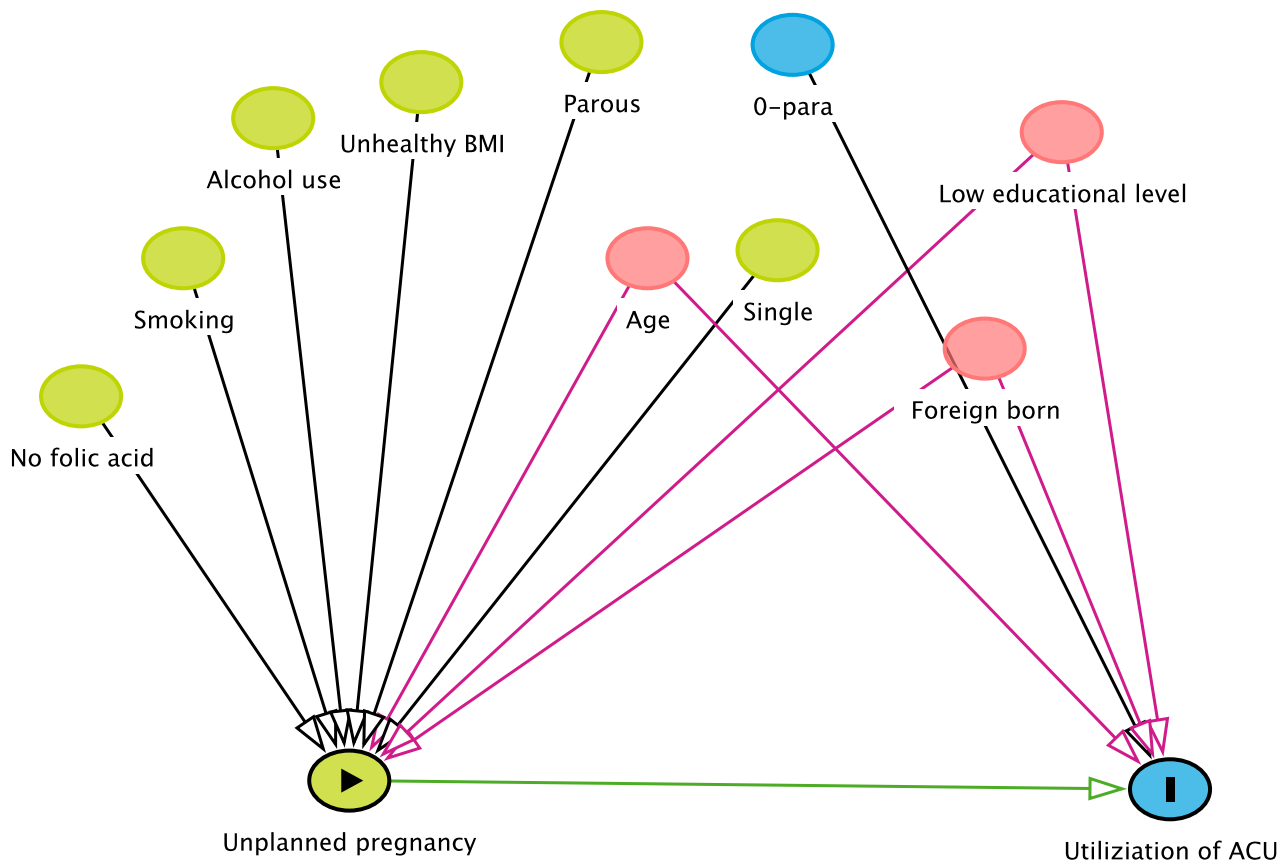

Supplement: S1 File — (PDF) [file pone.0286052.s001.pdf]

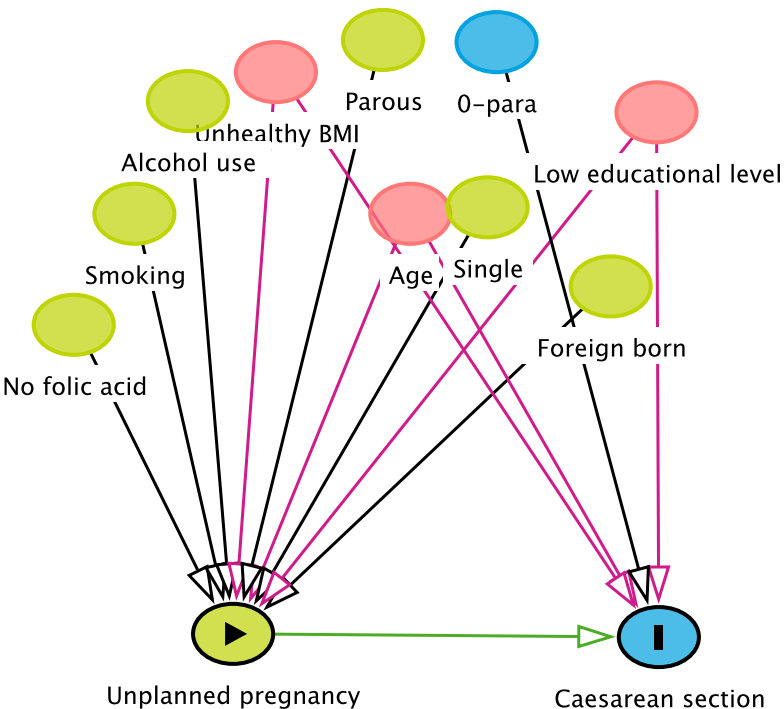

Supplement: S2 File — (PDF) [file pone.0286052.s002.pdf]

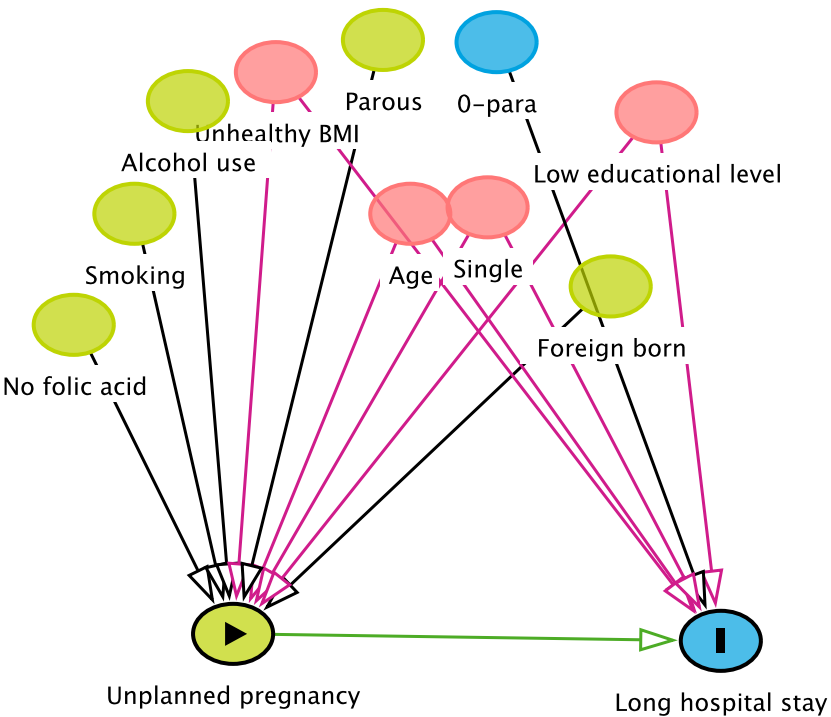

Supplement: S3 File — (PDF) [file pone.0286052.s003.pdf]

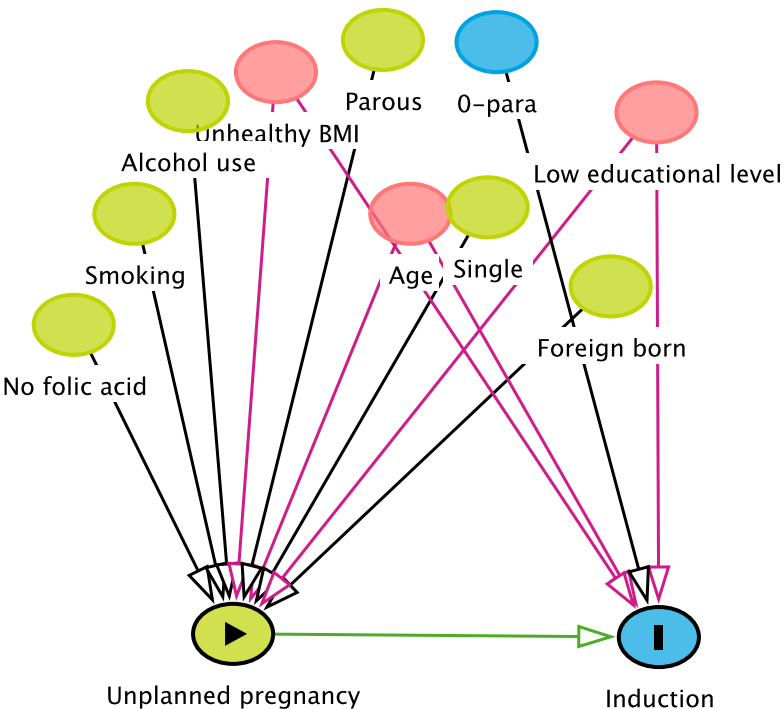

Supplement: S4 File — (PDF) [file pone.0286052.s004.pdf]

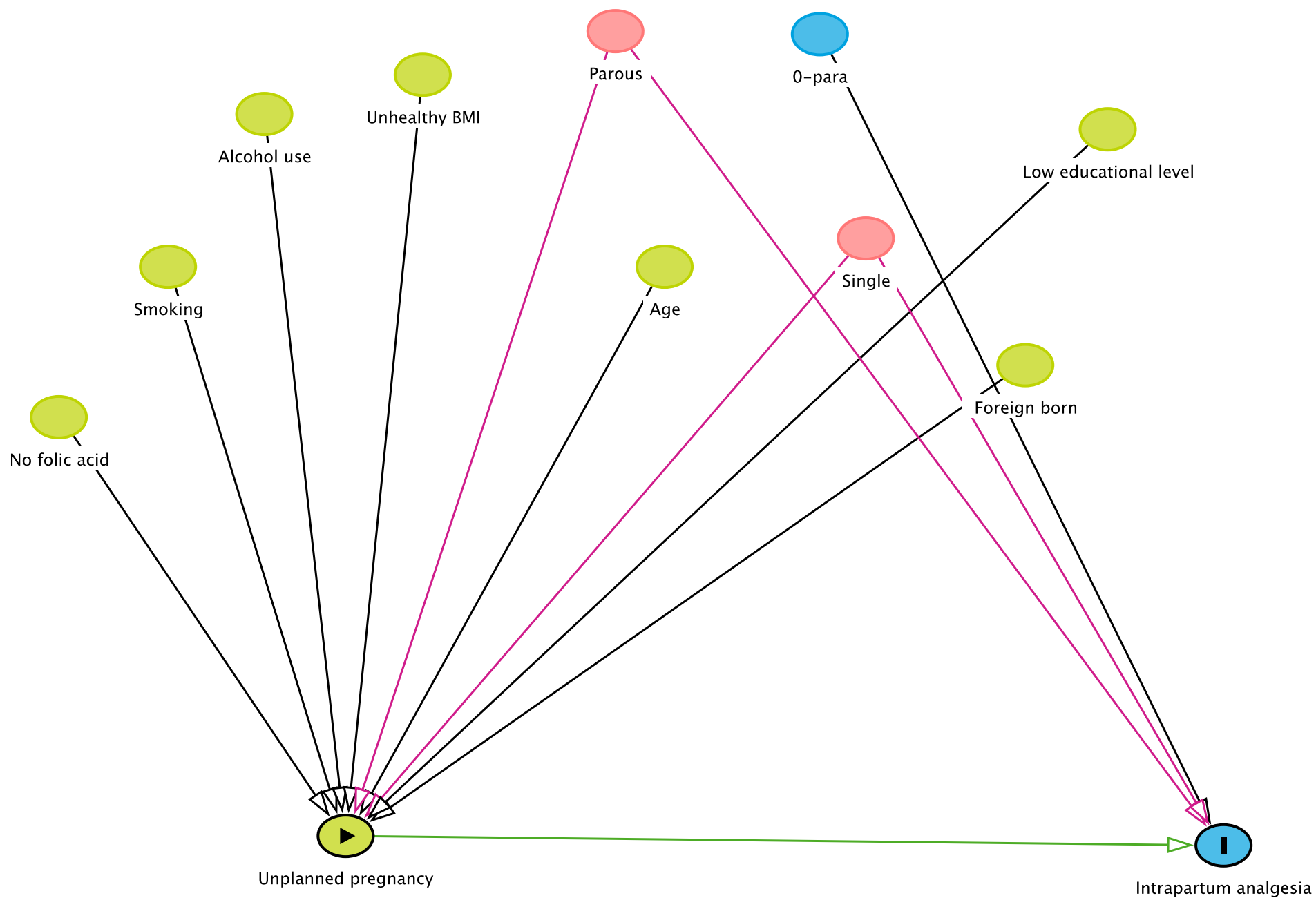

Supplement: S5 File — (PDF) [file pone.0286052.s005.pdf]

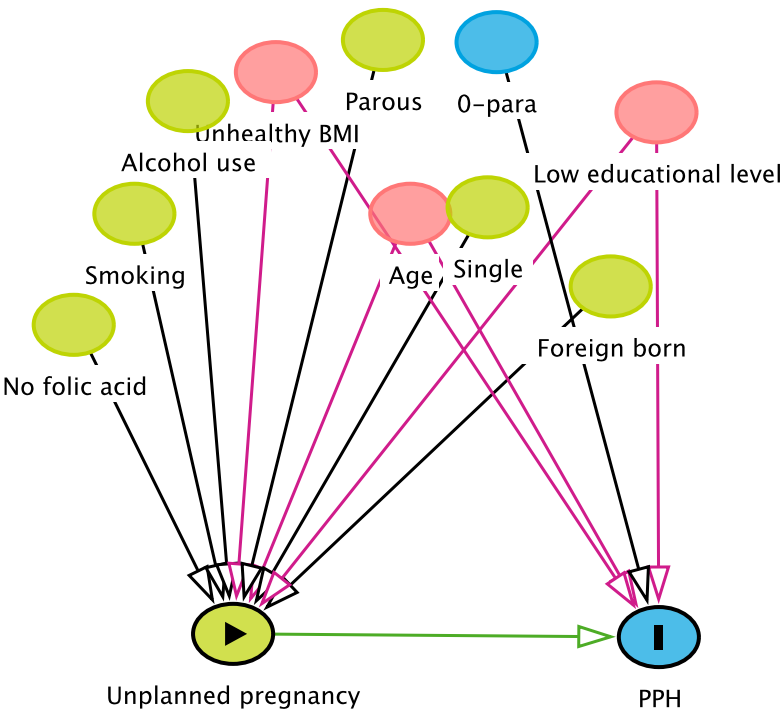

Supplement: S6 File — (PDF) [file pone.0286052.s006.pdf]

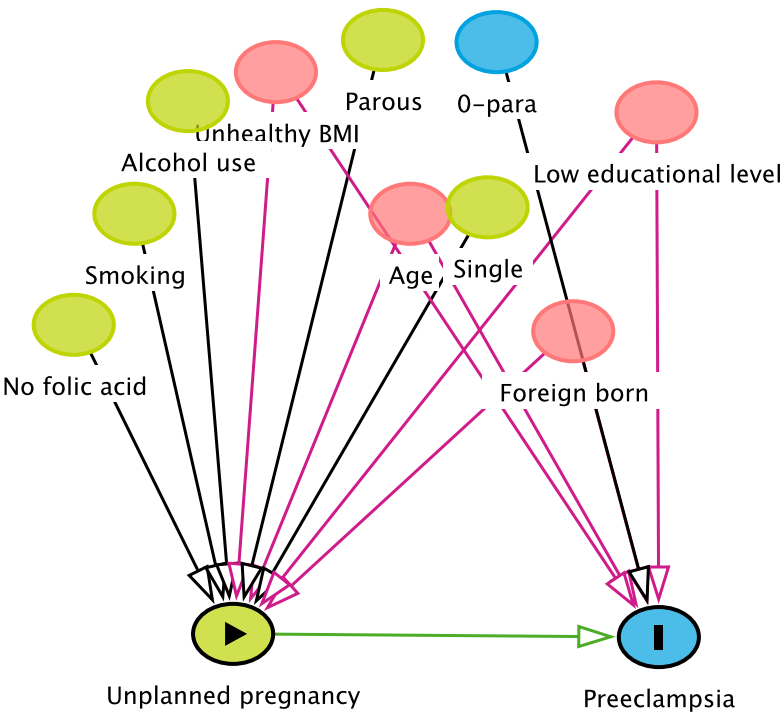

Supplement: S7 File — (PDF) [file pone.0286052.s007.pdf]

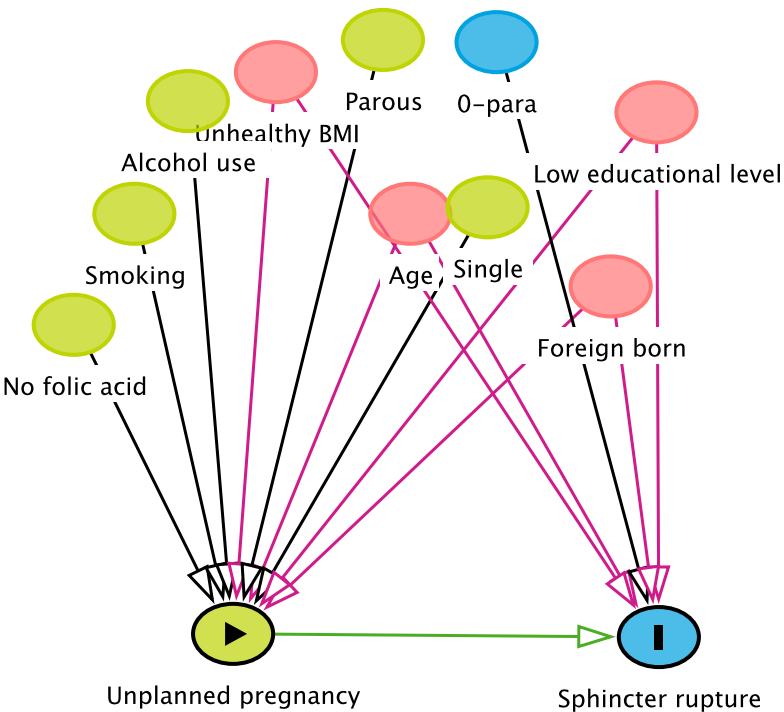

Supplement: S8 File — (PDF) [file pone.0286052.s008.pdf]

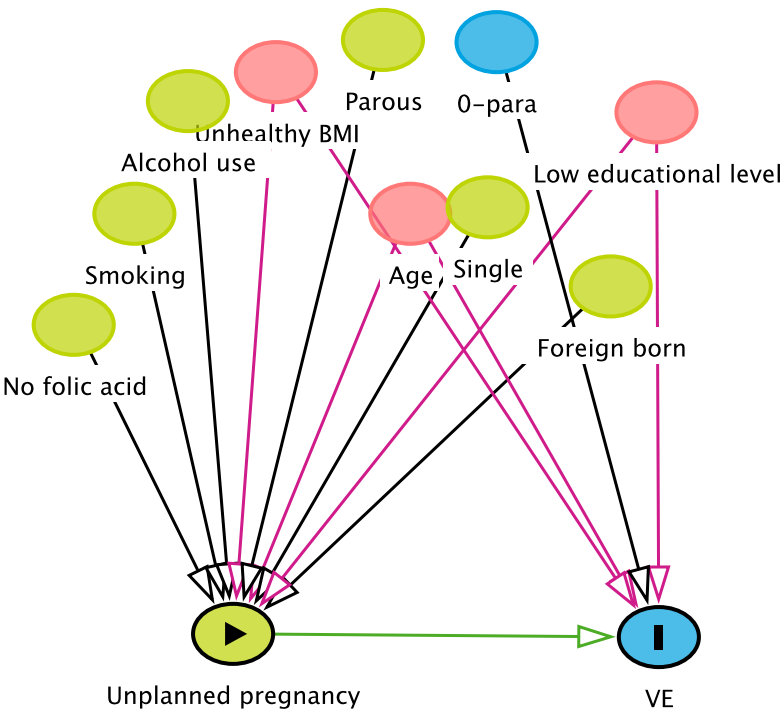

Supplement: S9 File — (PDF) [file pone.0286052.s009.pdf]
